# Efficient EPID‐based quality assurance of beam time delay for respiratory‐gated radiotherapy with validation on Catalyst™ and AlignRT™ systems

**DOI:** 10.1002/acm2.14376

**Published:** 2024-05-02

**Authors:** Kaining Yao, Meijiao Wang, Yi Du, Jiacheng Liu, Qingying Wang, Ruoxi Wang, Hao Wu, Haizhen Yue

**Affiliations:** ^1^ Key laboratory of Carcinogenesis and Translational Research (Ministry of Education/Beijing) Department of Radiation Oncology Peking University Cancer Hospital & Institute Beijing China; ^2^ Institute of Medical Technology Peking University Health Science Center Beijing China

**Keywords:** dose rate, quality assurance, respiratory‐gated radiotherapy, respiratory gating, time delay

## Abstract

**Purpose:**

To propose a straightforward and time‐efficient quality assurance (QA) approach of beam time delay for respiratory‐gated radiotherapy and validate the proposed method on typical respiratory gating systems, Catalyst™ and AlignRT™.

**Methods:**

The QA apparatus was composed of a motion platform and a Winston‐Lutz cube phantom (WL3) embedded with metal balls. The apparatus was first scanned in CT‐Sim and two types of QA plans specific for beam on and beam off time delay, respectively, were designed. Static reference images and motion testing images of the WL3 cube were acquired with EPID. By comparing the position differences of the embedded metal balls in the motion and reference images, beam time delays were determined. The proposed approach was validated on three linacs with either Catalyst™ or AlignRT™ respiratory gating systems. To investigate the impact of energy and dose rate on beam time delay, a range of QA plans with Eclipse (V15.7) were devised with varying energy and dose rates.

**Results:**

For all energies, the beam on time delays in AlignRT™ V6.3.226, AlignRT™ V7.1.1, and Catalyst™ were 92.13 ± 5.79 ms, 123.11 ± 6.44 ms, and 303.44 ± 4.28 ms, respectively. The beam off time delays in AlignRT™ V6.3.226, AlignRT™ V7.1.1, and Catalyst™ were 121.87 ± 1.34 ms, 119.33 ±0.75 ms, and 97.69 ± 2.02 ms, respectively. Furthermore, the beam on delays decreased slightly as dose rates increased for all gating systems, whereas the beam off delays remained unaffected.

**Conclusions:**

The validation results demonstrate the proposed QA approach of beam time delay for respiratory‐gated radiotherapy was both reproducible and time‐efficient to practice for institutions to customize accordingly.

## 1 INTRODUCTION

Respiratory motion is a major source of uncertainty in radiotherapy, as it can cause organ displacement and impair treatment accuracy.^1^ Respiratory gating techniques have been developed to mitigate this issue and several studies have shown the clinical benefits of using such techniques.^2^, ^3^, ^4^, ^5^, ^6^, ^7^ Respiratory gating techniques fundamentally require a synchronization of the radiation beam with the patient's respiratory cycle. The relationship between temporal accuracy and phase/amplitude gate used was established by gated treatment delivery. The time delay of respiratory gating systems may affect the accuracy of phase/amplitude gating, thereby affecting the accuracy of dose delivery.^8^ So, respiratory gating techniques have also presented new challenges for medical physicists, particularly in quality assurance (QA) work. To tackle these challenges, professional organizations have established task‐specific working groups, and several technical reports have been published as part of their efforts. For example, AAPM TG‐76^9^ provides a comprehensive guideline outlining general methods and tools for QA. AAPMTG‐142^8^ and AAPMTG‐198^10^ recommend a variety of items and reference standards for daily, monthly, and annual QA. These guidelines place significant emphasis on the importance of beam time delay QA in respiratory gating systems. Unfortunately, the reference QA methods mentioned in the reports may not provide adequate technical details for the implementation of reference QA methods. Early studies^11^, ^12^, ^13^ have investigated the beam time delay of RPM systems with comprehensive analysis. Therefore, Woods et al. recommend verifying the temporal accuracy of gating systems.^14^ However, the testing apparatus and data analysis tools were developed in‐house, making it hard for other institutions to reproduce their methods as QA program. Recently, Freislederer et al.^15^ proposed to the use of a 2D detector matrix to measure the beam on time delay of Catalyst™ gating system. However, their study did not include the measurement of beam off time delay. Li et al.^16^ developed a fitting‐based algorithm to determine the time delay of the Catalyst™ system. Barfield et al.^17^ utilized a commercial motion platform and EPID to investigate the time delay of AlignRT™ system. Naylor et al.^18^ and Stock et al.^19^ measured the movement distance of the marker block or film at different phantom velocities and then fitted the measurements with linear equations, of which the slope value represented the time delay. The studies mentioned above have certain limitations, including strict implementation requirements, complex data analysis procedures, limited universality, and challenges in comparing results across multiple platforms.

Our objective is to propose a precise and time‐efficient approach to measure both beam on and beam off time delays in respiratory‐gated radiotherapy. The detailed description of the apparatus, data preparation, image acquisition, and data analysis procedures were provided in the study. The used method was validated on two common respiratory gating systems: Catalyst™ (v5.4.2), AlignRT™(V6.3.226), and AlignRT™(v7.1.1). Furthermore, the impact of beam energy and dose rate on beam time delay was investigated and compared across these systems. Note that this work focuses on the time delay performance of these gating, and some systems are not yet licensed for respiratory gating (as per instructions for use).

## 2 MATERIALS AND METHODS

### 2.1 Characterization of motion devices

This study involved testing three types of respiratory gating systems: Catalyst™ from C‐RAD (V5.4.2) and AlignRT™ from VisionRT (V6.3.226 and V7.1.1). Two VitalBeam lincs were equipped with different AlignRT™ systems for real‐time monitoring during treatment delivery. These systems were capable of monitoring respiratory motion and real‐time tracking of the body surface. Their sampling frequency and camera parameters were suitable for clinical use according to Oh et al.^20^

**FIGURE 1:**
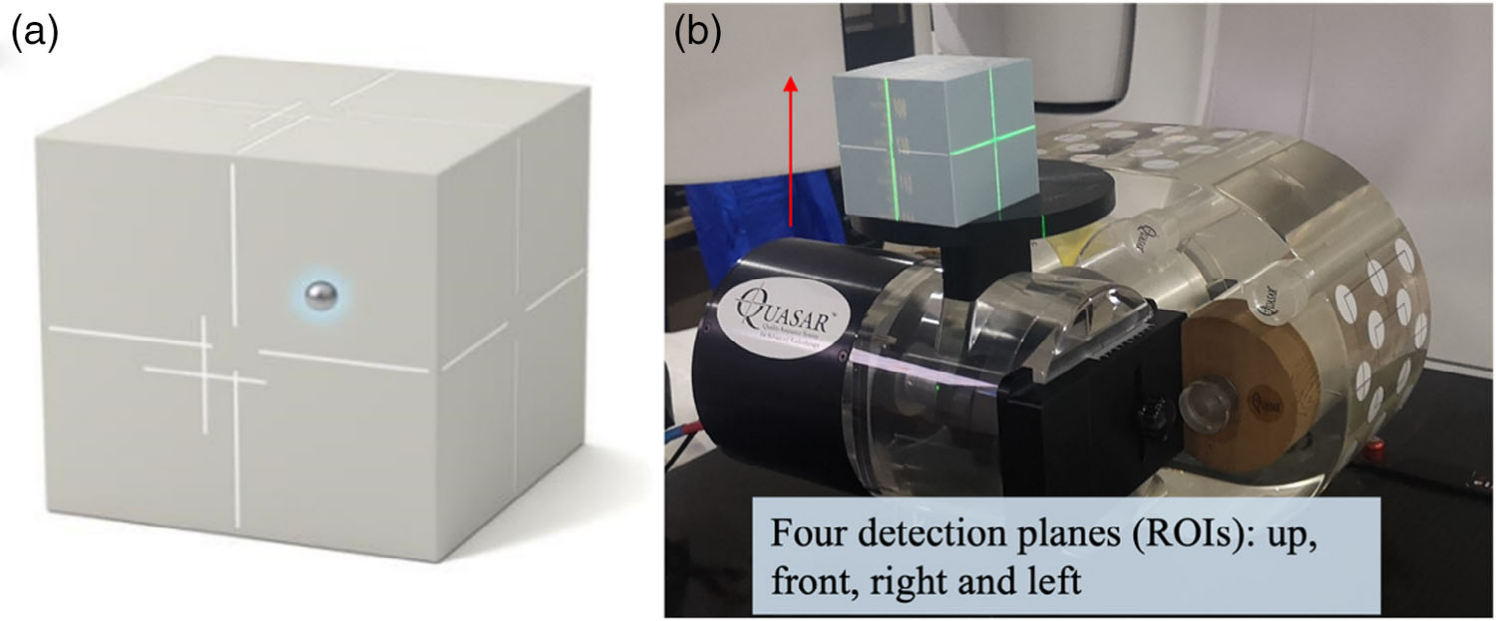
The setup of the QUASAR motion phantom and WL3 cube. (a) shows the WL3 cube. (b) shows phantom's setup and four detection planes. The red arrow shows the movement direction of phantom.

We utilized the QUASAR programmable respiratory motion phantom from Modus Medical Devices (London, Canada) to simulate regular respiratory waveforms.^21^ We performed annual quality control on the motion phantom with ruler and stopwatch. The amplitude and frequency of the phantom's motion could be adjusted programmatically. To visualize and monitor the motion using the Catalyst™ and AlignRT™ systems, the WL3 cube (Mobius Medical System, LP, Houston, TX) was attached to the QUASAR phantom. The WL3 cube featured a marked line on its surface, which facilitated accurate setup. Additionally, it contained a built‐in metal ball that produced a high‐contrast EPID image (see Figure 1a). This metal ball was used for analyzing position coordinates during the analysis process.

### 2.2 Plan preparation

Once the WL3 cube was attached to the QUASAR motion phantom, the combined motion phantom was scanned using a Siemens Sensation Open CT scanner. The QUASAR software was used to control the speed, amplitude and frequency of the combined motion phantom. The generated respiratory waveform was saved to ensure that the motion could be precisely replicated during subsequent testing on the linac.

In the Eclipse planning system *(version 15.6)*, two types of plans were developed based on CT images: one for measuring beam on time delay and the other for measuring beam off time delay. To measure the beam on time delay, a plan with the minimal beam delivery, that is, 1 monitor unit (MU), was designed to acquire a snapshot image of the motion phantom. To measure the beam off time delay, a beam with constant dose rate at the end of the radiation delivery was necessary. Therefore, the planned MU was determined based on the energy and dose rate settings, typically set above 100 MU.

Plans were designed to assess the impact of energy and dose rate on time delay: 10xFFF (2400 MU/min), 6xFFF (400, 600, 1000, 1200, and 1400 MU/min), 6x (600 MU/min), 10x (400 MU/min). These plans were used to compare time delay between different linacs.

### 2.3 Images acquisition

#### 2.3.1 Acquisition of motion images

**FIGURE 2:**
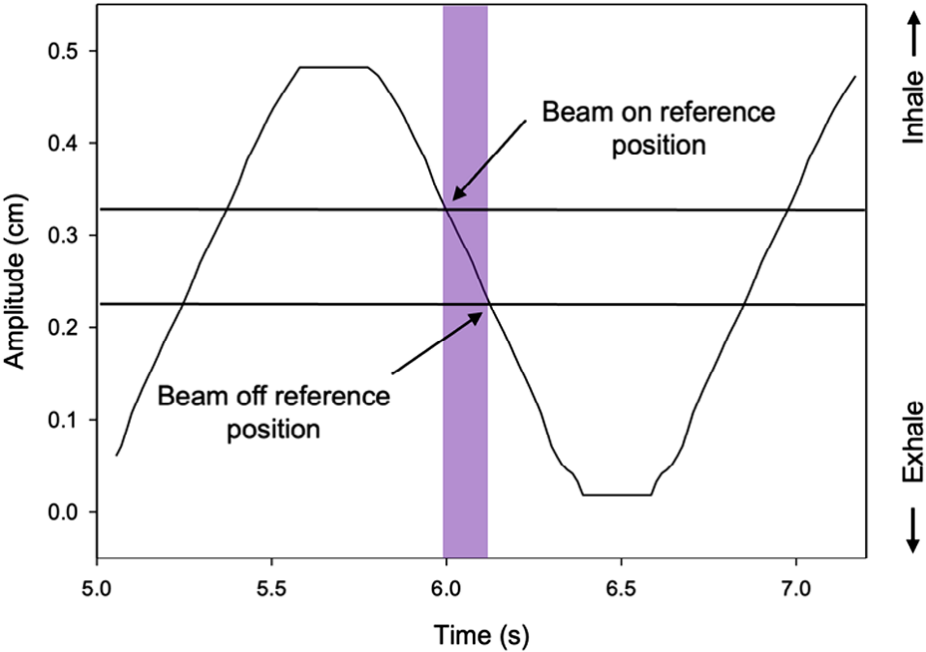
The respiratory waveform of motion phantom in QUASAR software. Purple bar indicates beam‐on time. The black arrows indicate the reference positions of beam on and beam off.

Motion images were captured using EPIDs installed on a Varian EDGE and different VitalBeam (VB) linacs. The imager type of EPIDs were DMI, which have a size of 43 cm × 43 cm and a high resolution of 0.34 mm. Beam source to imager distance was 160 cm. The QUASAR motion phantom and WL3 cube moved with a consistent velocity. Figure 1b showed the motion phantom and WL3 cube setup and the detected ROIs used in the respiratory gating systems. Figure 2 showed the respiratory waveform of motion phantom in QUASAR software which included the reference position of beam on and beam off. Ideally, the positions of metal ball in the motion images and reference images are basically overlap. In actual scenario, due to the inherent time delay in respiratory gating systems, the positions of the metal ball in the motion image and reference image are mismatched. The motion images were acquired at a gantry angle of 90 degrees to ensure that the x‐ray direction to EPID is perpendicular to the motion direction of the WL3 cube. Plans were executed five times to reduce errors in analysis.

#### 2.3.2 Acquisition of reference images

**FIGURE 3:**
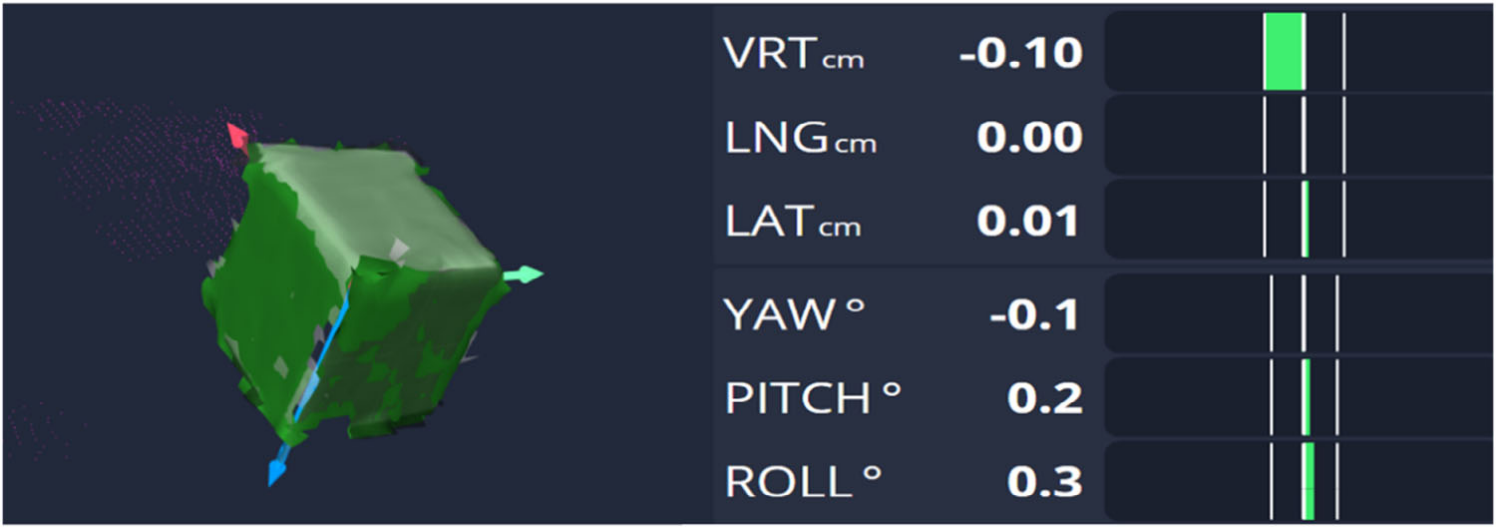
The gating window for AlignRT™ systems. The cube moves in the vertical direction.

**FIGURE 4:**
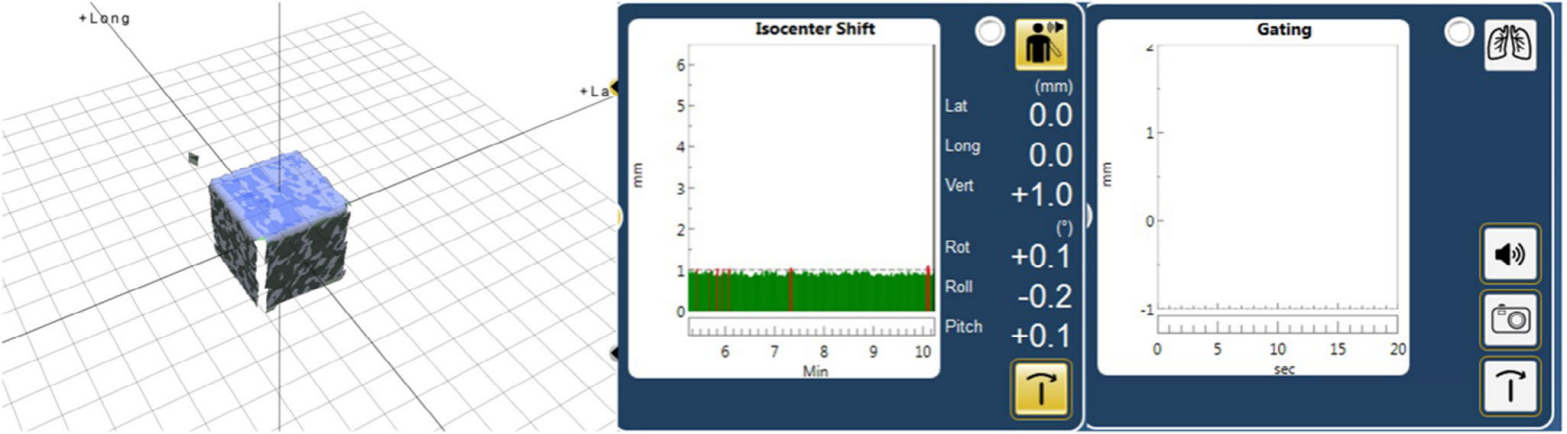
The gating window for Catalyst™ systems. The cube moves in the vertical direction.

The Catalyst™ and AlignRT™ systems detected the surface of the WL3 cube directly. Each respiratory gating system needed to set a detection range, within which beam‐on is allowed, which is the gating window. Figures 3 and 4 show the gating window for two systems. Gating window is an important parameter of respiratory gating systems. Considering the size of the phantom and the range of the time delay, a gating window of ± 1 mm was set. The QUASAR motion phantom and WL3 cube moved in respiratory waveform, the MU was delivered when the respiratory gating system detected the cube motion within a range of ± 1 mm from the reference position. The reference image acquisition location is fixed and static, assuming a time delay of 0. It is important to note, to take into account phantom motion direction, the reference phantom positions of beam on and beam off are not the same. For example, when the phantom moved from 20 to 0 mm (as shown in Figure 2), the reference position of beam on was at 2 mm, while that for beam off was at 0 mm in respiratory gating systems. Static reference images were acquired at a gantry angle of 90 degrees five times.

### 2.4 Calculation of beam time delay

#### 2.4.1 Calculation of beam on time delay

**FIGURE 5:**
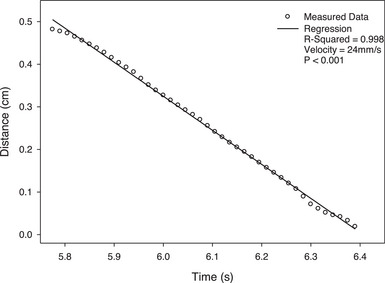
The motion fitted curve of the QUASAR motion phantom and WL3 cube. This is a part of the respiratory waveform. The *R*‐Squared of 0.998 demonstrates that the motion phantom and WL3 cube move uniformly with a strong correlation coefficient. The velocity of motion phantom and WL3 cube is 24 mm/s. A *p*‐value of less than 0.05 indicates that the fitted line has statistical significance.

**FIGURE 6:**
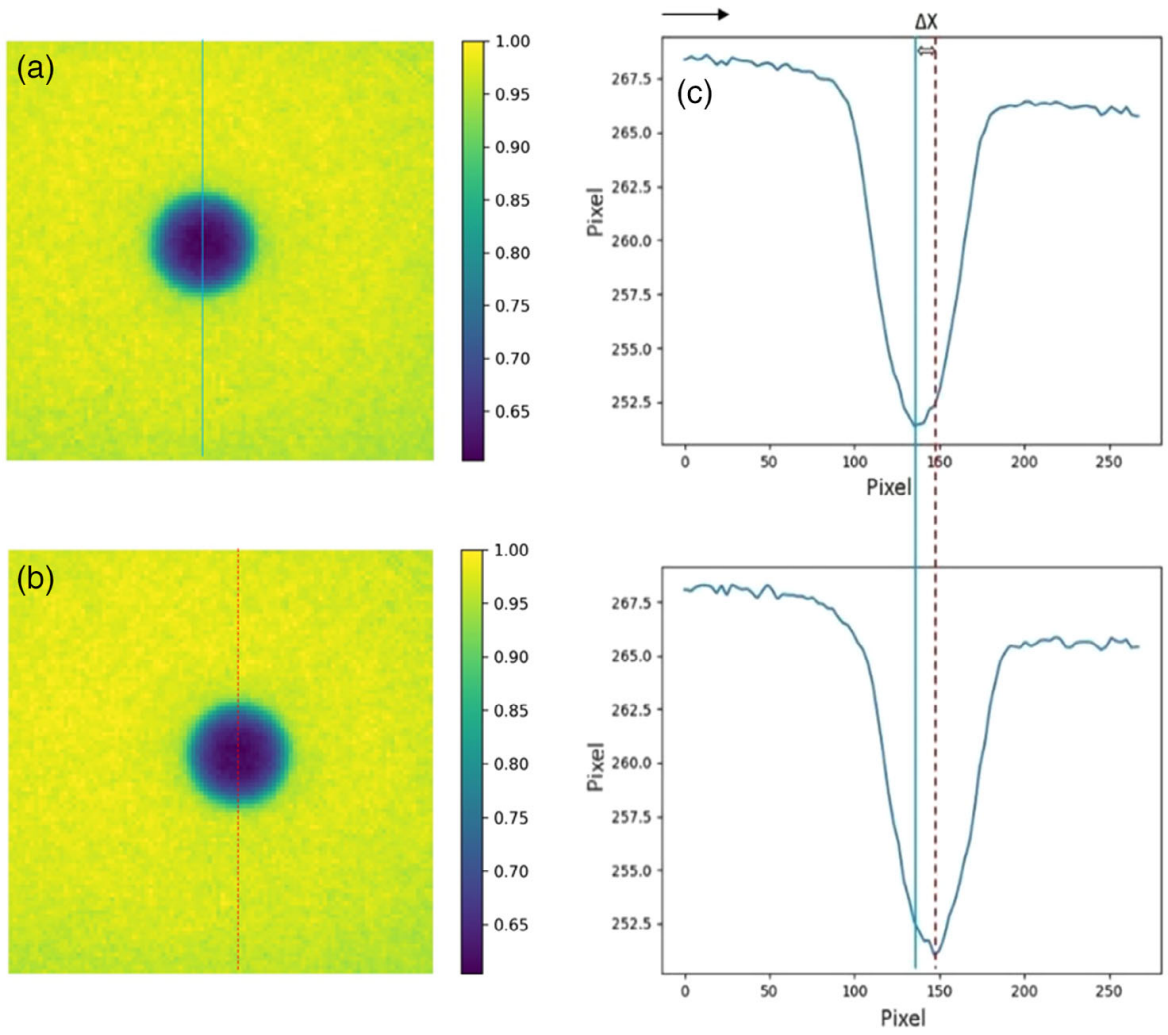
The metal ball position discrepancy between motion and reference images for beam on time delay calculation. (a) shows reference image, (b) shows motion image, (c) shows comparative analysis of reference and motion image profiles. The black arrow indicates the direction of motion, the blue solid line represents the center of the metal ball in the reference image and the red dotted line represents the center of the metal ball in the motion image. Δ X indicates the position discrepancy of metal ball.

**FIGURE 7:**
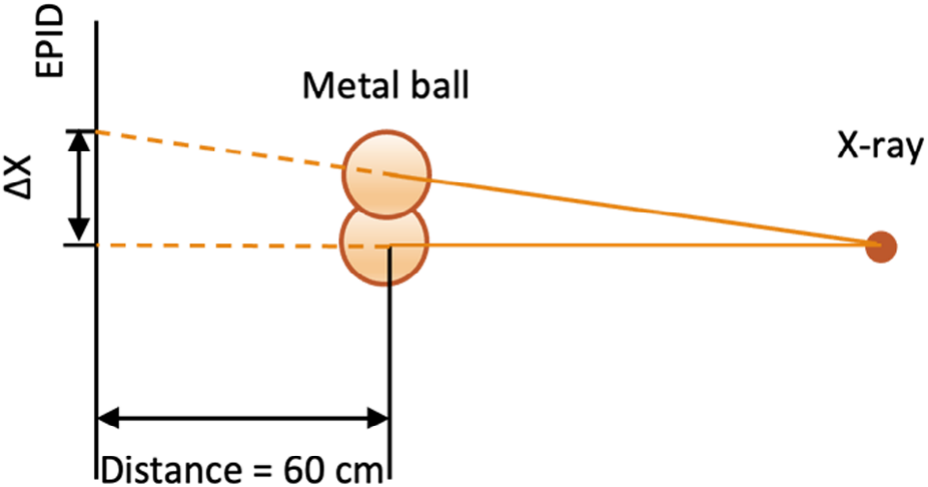
Geometric relationship between EPID extending distance and metal ball projection size. Δ X indicates the position discrepancy of metal ball. Distance means the source from metal ball to detector.

Considering the respiratory waveform coming from QUASAR software, it was essential to accurately simulate the movement velocity of the QUASAR motion phantom. The velocity was 24 mm/s as per the motion fitted curve (see the Figure 5), which was used to calculate beam delay time. In Figure 6, the distance between the metal ball in the motion image and reference image is utilized to calculate the beam on time delay. The extending distance of EPID does not affect the results. As shown in the Figure 7, the geometric relationship between ΔX and distance is similar triangles. The calculation formula for time delay is as follows:

(1) $$t=\frac{\Delta x}{v}$$

(2) $$\Delta x=P_{meas}-P_{ref}$$

where *t* represents time delay, *v* represents velocity of the QUASAR motion phantom, and *Δx* represents the position discrepancy of the metal ball. *P_meas_* represents the position of the metal ball in the motion image, while *P_ref_* represents the position of the metal ball in the reference image. The image analysis and peak position extraction to determine time delays was performed using an in‐house Python script.

#### 2.4.2 Calculation of beam off time delay

**FIGURE 8:**
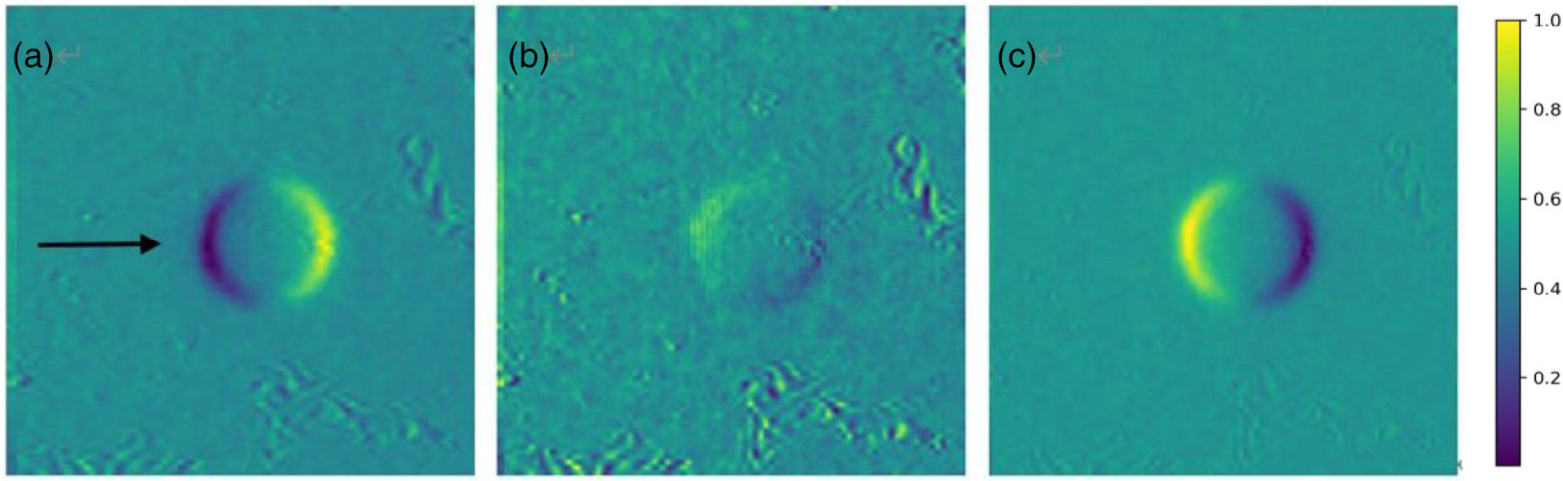
The metal ball position discrepancy between motion and reference images for beam off time delay calculation: (a) represents the motion image on the left side of the reference image, (b) demonstrates that the metal balls in the motion and reference images align perfectly, (c) represents the motion image on the right side of the reference image. The arrow indicates the direction of motion.

Since the metal ball in the image captured at the end of the beam appears as an ellipse, encompassing the motion trajectory, specific analysis methods different from that of beam on have been developed to address this. During the image analysis process, the metal ball in the reference image was intentionally displaced toward the direction of the QUASAR motion phantom by a distance. We used different pixels ball to find the stopping position of the metal ball according to a distance. The motion trajectory of the ball varies with different energies. Therefore, a distance is not just a single value, but a representation of a series of values. When the metal ball in the reference and motion images aligned perfectly (as shown in Figure 8b), the precise stop position of the motion image was determined, and the distance of the metal ball was calculated. The beam off time delay was subsequently calculated, considering the velocity of the phantom. The calculation formula for the beam off time delay is same as that used for the beam on time delay.

## 3 RESULTS

### 3.1 Beam on time delay of Catalyst™ system

The results obtained from the Catalyst™ system installed on the EDGE revealed small variations in time delay for each energy, which are presented in Table 1. Mean distance discrepancy (referred to as MDD) indicates average Δx over five repeated measurements. The time delays for beam of 10xFFF, 6xFFF, 6x, and 10x were recorded as 297.08, 302.50, 305.42, and 308.75 ms respectively. This may be due to the long optical detection time of the Catalyst™ system itself.

**TABLE 1:** Beam on time delay of Catalyst™ system.

		Time delay (ms)	
Beam mode	MDD (mm)^a^	Mean	SD^b^
10xFFF, 2400	7.13	297.08	14.17
6xFFF, 1200	7.26	302.50	6.67
6x, 600	7.33	305.42	5.42
10x, 400	7.41	308.75	20.00

^a^ MDD is average Δx over five repeated measurements.

^b^ SD is standard deviation over five repeated measurements.

### 3.2 Beam off time delay of Catalyst™ system

During the measurement of beam off time delay for the Catalyst™ system, we used beam‐on‐off mode and Table 2 displayed the results. The table indicated that the beam off time delay difference for each energy on the Catalyst™ was small and consistent. The time delays for beam of 10xFFF, 6xFFF, 6x, and 10x were recorded as 97.69, 97.07, 100.80, and 95.20 ms respectively.

**TABLE 2:** Beam off time delay of Catalyst™ system.

		Time delay (ms)	
Beam mode	MDD (mm)	Mean	SD
10xFFF, 2400	2.35	97.69	1.87
6xFFF, 1200	2.33	97.07	1.87
6x, 600	2.42	100.80	2.29
10x, 400	2.29	95.20	2.29

### 3.3 Beam on time delay of AlignRT™ systems

About two versions of AlignRT™ systems (V6.3.226 and V7.1.1), the measured beam on time delay of different energies using these systems is summarized in Table 3, where the time delay for different version of AlignRT™ systems was found to be slightly different. Specifically, the time delay for V6.3.226 exhibited a range of 85–100 ms but V7.1.1 exhibited 112–130 ms.

**TABLE 3:** Beam on time delay of two version AlignRT™ systems.

	AlignRT™ V6.3.226			AlignRT™ V7.1.1		
Beam mode	MDD (mm)	Time delay (ms)	SD	MDD (mm)	Time delay (ms)	SD
10xFFF, 2400	2.04	85.09	13.43	2.24	93.34	17.41
6xFFF, 1200	2.12	88.39	13.98	3.04	126.55	11.08
6x, 600	2.28	95.04	17.09	3.10	129.10	18.11
10x, 400	2.40	100.00	22.37	2.99	124.44	5.09

### 3.4 Beam off time delay of two version AlignRT™ systems

The beam off time delay of AlignRT™ system was measured in on‐off mode. As shown in Table 4, the results exhibited a high level of consistency in time delays across different energy. For beam modes of (10xFFF,2400), (6xFFF1200), (6 × 600), and (10 × 400), the time delay of V6.3.226 were 121.80, 123.41, 122.50, and 119.78 ms, while V7.1.1 were 119.33, 120.67, 120.00, and 118.67 ms. The time delay between the two versions displayed a generally consistent relationship.

**TABLE 4:** Beam off time delay of two version AlignRT™ systems.

	AlignRT™ V6.3.226			AlignRT™ V7.1.1		
Beam mode	MDD (mm)	Time delay (ms)	SD	MDD (mm)	Time delay (ms)	SD
10xFFF, 2400	2.92	121.80	1.40	2.86	119.33	2.31
6xFFF, 1200	2.96	123.41	2.32	2.90	120.67	1.63
6x, 600	2.94	122.50	2.02	2.88	120.00	2.11
10x, 400	2.88	119.78	2.20	2.85	118.67	2.31

### 3.5 Dose rate related beam time delay

Considering the sensitivity of measurement, AlignRT™ system with the shortest response time was selected to study the influence of dose rate on beam time delay in detail. Due to the minimal variation in beam time delay between the two versions of the AlignRT™ system, only AlignRT™ V7.1.1 was utilized for the implementation of the 6xFFF plans. Table 5 demonstrates a significant trend: in the 6 FFF energy mode, except for 400 MU/min, the beam on time delay decreases with the increase of dose rate. However, the beam off time delay does not demonstrate a similar characteristic, as the beam off time delay remains relatively consistent across different dose rates.

**TABLE 5:** Beam on and off time delay of two version AlignRT™ systems.

	Beam on			Beam off		
	MDD (mm)	Time delay (ms)	SD	MDD (mm)	Time delay (ms)	SD
6xFFF, 400	3.32	138.29	0.29	2.92	121.60	1.87
6xFFF, 600	3.40	141.63	7.27	2.92	121.60	1.87
6xFFF, 1000	3.28	136.86	7.00	2.87	119.50	2.02
6xFFF, 1200	3.23	134.71	6.61	2.84	118.33	2.33
6xFFF, 1400	3.04	126.52	0.39	2.86	119.11	2.20

## 4 DISCUSSION

In this study, we proposed a precise and time‐efficient method for measuring beam on and off time delay of three respiratory gating systems (Catalyst™, AlignRT™ V6.3.226, and AlignRT™ V7.1.1) and investigated the effects of energy and dose rate on beam time delay. The results demonstrate variations in beam on time delay across different systems, energies, and dose rates. But no significant difference was found between the two versions of AlignRT™. On the other hand, beam off time delay exhibited consistency with energy and dose rate but revealed differences between the Catalyst™ and AlignRT™ systems. These findings indicate the need to establish customized QA reference levels for beam time delay in different respiratory gating systems to meet clinical requirements. We have noticed that there are similar works about beam time delay. However, compared with these studies, our work in time delay measurement is kind of different. Firstly, we used different tools (QUASAR motion phantom and WL3 cub). Secondly, the algorithm in calculating beam‐off delay is different.

For the Catalyst™ system, the beam on time delay for each energy was less than 310 ms and the beam off time delay was less than 101 ms. This may be due to the long response time and insufficient stability of the system. It is a characteristic of the system and is independent of the experimental design. In the study conducted by Li et al.,^16^ the beam on time delay for the EDGE was 303 ± 45 ms and the beam off time delay was 34 ± 25 ms, which is basically consistent with our findings. However, Li et al. set a wider region of interest, which may have contributed to discrepancies. Li's study also showed that the longer the beam hold time (409–4264 ms) of the Catalyst™ system, the higher the beam on time delay. The time delay measured in our work is consistent with the previous work (303.44 ms herein vs. 303 ms^16^ for beam on time delay and 121.87 ms herein vs. 215 ms^15^ for beam off time delay).

The beam on time delays in AlignRT™ V6.3.226 and AlignRT™ V7.1.1 are 92.13 ± 5.79 ms and 123.11 ± 6.44 ms, respectively. However, the consistency of beam off time delay for each energy was high in both versions, and the maximum time delay discrepancy is 4.74 ms. This phenomenon can be explained by the fact that when MU is sufficient, the time required for the dose rate of the Varian linac to reach the expected value and then decrease from the expected value to zero is almost the same. Due to the plan for beam on only have 1MU, the dose rate may not reach the expected value in an instant. The fluctuation of dose rate will cause SD to be larger. This can also explain why the SD of beam on is larger than that of beam off. The beam on time delay measured in our work is consistent with the previous work (120 ms herein vs. 170 ms,^18^ >200 ms^17^).

In this study, we also used 6xFFF with more dose rates to investigate the influence of dose rate on beam time delay. The results showed that except for 400 MU/min, the beam on time delay decreases with the increase of dose rate in AlignRT™V7.1.1. However, it is worth noting that the variation in time delay is slightly greater. The beam off time delay remained consistent across different energies and was not significantly influenced by changes in dose rate. This was observed as the dose rate decreased from a predetermined value to zero.

In this study, the velocity of motion phantom is 24 mm/s. The reason we used a special waveform is to set up a reference condition, which is highly reproducible to facilitate regular QA activities. But we agree that different velocities may result in different time delay. And we will study on different velocities in the future.

The limitation of the proposed method is that we only measured the end‐to‐end beam time delay over the entire respiratory gated beam delivery rather than time delays in any sub‐processes. From the perspective of beam delivery, we believe this end‐to‐end time delay is of key interest in time delay QA for respiratory gating. Additionally, we selected eight typical beam modes for method illustration purposes in this study and the results were cross‐sectional in nature. Future work will focus on analyzing the long‐term trends of the Catalyst™ and AlignRT™ gating systems.

## 5 CONCLUSION

This study proposed a precise and time‐efficient method for measuring the beam on and off time delays on three types of respiratory gating systems. This is the first study to measure the time delays of Catalyst™ (V5.2.4), AlignRT™ (V6.3.226), and AlignRT™ (V7.1.1) systems using the same method. The validation results indicate that the proposed QA method of beam time delay was reproducible and efficient as routine clinical QA practice.

## AUTHOR CONTRIBUTIONS

Kaining Yao and Meijiao Wang drafted the manuscript and contributed equally to this paper. Jiacheng Liu and Qingying Wang performed experiments and data analysis. Ruoxi Wang and Hao Wu designed the study. Hao Wu performed data analysis. Haizhen Yue was the Sub‐I and Yi Du was the PI.

## CONFLICT OF INTEREST STATEMENT

The authors declare that they have no competing interests.

## CONSENT FOR PUBLICATION

All the authors reviewed the manuscript and approved the final submission to Radiation Oncology.

## Data Availability

The data that support this study are not open access but are available from the corresponding author upon reasonable request.
